# ICTV Virus Taxonomy Profile: *Geminiviridae*

**DOI:** 10.1099/jgv.0.000738

**Published:** 2017-03-13

**Authors:** F. Murilo Zerbini, Rob W Briddon, Ali Idris, Darren P Martin, Enrique Moriones, Jesús Navas-Castillo, Rafael Rivera-Bustamante, Philippe Roumagnac, Arvind Varsani, ICTV Report Consortium

**Affiliations:** ^1^​ Departamento de Fitopatologia/Bioagro, Universidade Federal de Viçosa, Viçosa, Minas Gerais 36570-900, Brazil; ^2^​ National Institute for Biotechnology and Genetic Engineering, Jhang Road, P.O. Box 577, Faisalabad, Pakistan; ^3^​ School of Plant Sciences, University of Arizona, Tucson, AZ 85721-0107, USA; ^4^​ Computational Biology Group, Institute of Infectious Diseases and Molecular Medicine, University of Cape Town, Cape Town, South Africa; ^5^​ Instituto de Hortofruticultura Subtropical y Mediterránea ‘La Mayora’, Universidad de Málaga-Consejo Superior de Investigaciones Científicas (IHSM-UMA-CSIC), 29750 Algarrobo-Costa, Málaga, Spain; ^6^​ Departamento de Ingeniería Genética, Centro de Investigación y de Estudios Avanzados del IPN (Cinvestav) – Unidad Irapuato, 36821 Irapuato, GTO, Mexico; ^7^​ CIRAD-INRA-SupAgro, UMR BGPI, Campus International de Montferrier-Baillarguet, 34398 Montpellier Cedex-5, France; ^8^​ The Biodesign Center for Fundamental and Applied Microbiomics, School of Life Sciences, Center for Evolution and Medicine, Arizona State University, Tempe, AZ 85287, USA

**Keywords:** *Geminiviridae*, ICTV Report, taxonomy

## Abstract

The geminiviruses are a family of small, non-enveloped viruses with single-stranded, circular DNA genomes of 2500–5200 bases. Geminiviruses are transmitted by various types of insect (whiteflies, leafhoppers, treehoppers and aphids). Members of the genus *Begomovirus* are transmitted by whiteflies, those in the genera *Becurtovirus*, *Curtovirus*, *Grablovirus, Mastrevirus* and *Turncurtovirus* are transmitted by specific leafhoppers, the single member of the genus *Topocuvirus* is transmitted by a treehopper and one member of the genus *Capulavirus* is transmitted by an aphid. Geminiviruses are plant pathogens causing economically important diseases in most tropical and subtropical regions of the world. This is a summary of the International Committee on Taxonomy of Viruses (ICTV) Report on the taxonomy of the *Geminiviridae* which is available at www.ictv.global/report/geminiviridae.

## Virion

**Fig. 1.**
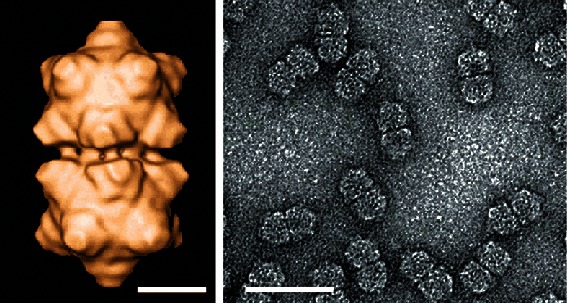
(Left) Cryo-electron microscopic reconstruction of maize streak virus viewed along a two-fold axis of symmetry. Bar, 10 nm. (Right) Purified particles of maize streak virus stained with uranyl acetate showing typical twinned quasi-isometric subunits. Bar, 50 nm. (From [1]; courtesy of R. McKenna.)

Geminiviruses have a unique particle morphology of twinned (geminate) icosahedra. For maize streak virus (genus *Mastrevirus*), virions are 22×38 nm, consisting of two incomplete icosahedra (T=1) containing 110 coat protein subunits organized as 22 pentameric capsomers (Table 1 and Fig. 1) [1].

**Table 1.** Characteristics of the family *Geminiviridae*

Typical member:	bean golden yellow mosaic virus-[Dominican Republic:1987] (DNA-A: L01635; DNA-B: L01636), species *Bean golden yellow mosaic virus*, genus *Begomovirus*
Virion	Twinned (geminate) incomplete icosahedra, T=1, 22×38 nm with a single coat protein
Genome	2.5–5.2 kb of single-stranded, circular DNA, mono- or bipartite
Replication	Complementary strand synthesized in the nucleus by host replication factors; double-stranded circular molecules serve as templates for both transcription and replication; replication employs a rolling-circle mechanism and also a recombination-dependent mechanism
Translation	From transcribed mRNAs
Host range	Plants (monocots and dicots)
Taxonomy	Nine genera collectively containing >360 species

## Genome

**Fig. 2.**
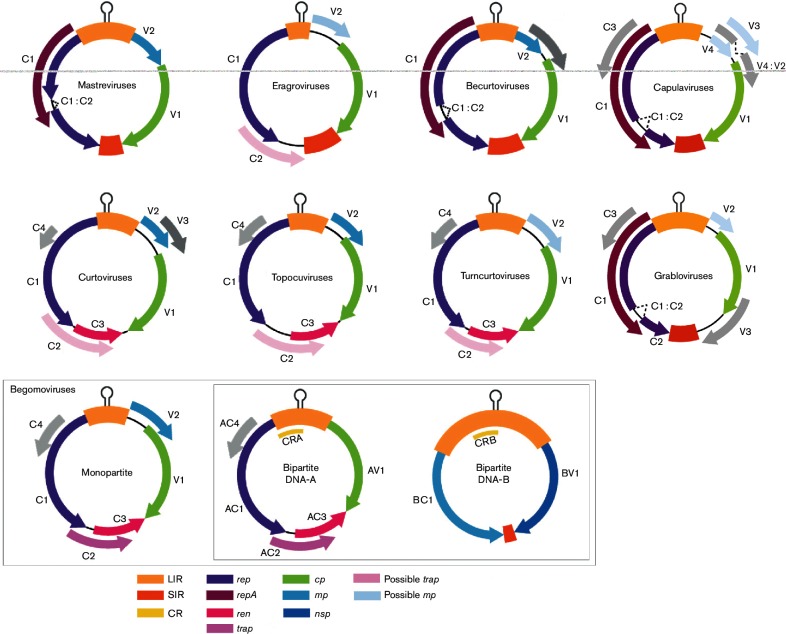
Genome organization of isolates in various geminivirus lineages. The ORFs (V1, V2, V3, C1, etc.) are colour-coded according to the function of their protein products (rep, replication-associated protein; ren, replication enhancer protein; trap, transcriptional activator protein; cp, capsid protein; mp, movement protein; nsp, nuclear shuttle protein). LIR, long intergenic region; SIR, short intergenic region; CR, common region. The hairpin which includes the origin of replication is indicated in the LIR (modified from [4]).

Viruses in the genera *Becurtovirus*, *Capulavirus*, *Curtovirus*, *Eragrovirus*, *Grablovirus*, *Mastrevirus*, *Topocuvirus* and *Turncurtovirus* have monopartite genomes, whereas those in the genus *Begomovirus* have mono- or bipartite genomes. The genome of mastreviruses (Fig. 2) consists of a circular single-stranded DNA of 2.6–2.8 kb that encodes a capsid protein (CP, ORF V1), a movement protein (MP, ORF V2) and a replication-associated protein (Rep, expressed from ORFs C1 and C2 by transcript splicing). The genomes of bipartite begomoviruses consist of DNA-A and DNA-B components, each of 2.5–2.6 kb. The two components share approximately 200 bases of sequence within the long intergenic region (LIR) that includes the replication origin. DNA-A encodes CP (AV1/V1), a putative MP (AV2/V2; New World bipartite viruses lack AV2), Rep (AC1/C1), a transcriptional activator (TrAP, AC2/C2), a replication enhancer (REn, AC3/C3) and C4 (AC4/C4). DNA-B encodes a nuclear shuttling protein (NSP, BV1) and MP (BC1). The genomes of monopartite begomoviruses resemble the bipartite DNA-A component [2].

## Replication

Complementary-sense DNA synthesis to produce dsDNA depends solely on host factors. Virus ssDNA synthesis is initiated by cleavage of the virion-sense strand by Rep in a conserved 5′-TAATATTAC-3′ sequence within the LIR. Geminiviruses do not encode a DNA polymerase, relying on host factors recruited during the early stages of replication. Coding regions in both strands diverge from the LIR, and transcription is bi-directional. Geminiviruses use multiple overlapping transcripts for gene expression [3].

## Taxonomy

### Becurtovirus

This genus contains two species, *Beet curly top Iran virus* and *Spinach curly top Arizona virus* [4]. Members are transmitted by leafhoppers to dicot plants.

### Begomovirus

This genus consists of >320 species. Begomoviruses infect dicots and are transmitted by whiteflies [5]. Most monopartite begomoviruses are associated with DNA satellites. Important pathogens include members of the species *African cassava mosaic virus*, *Bean golden mosaic virus*, *Cotton leaf curl Kokhran virus* and *Tomato yellow leaf curl virus*.

### Capulavirus

This genus contains four species. Isolates of the species *Alfalfa leaf curl virus* are transmitted by an aphid [6].

### Curtovirus

This genus contains three species including *Beet curly top virus*, members of which are important pathogens in North America and Iran [7]. Members infect dicots and are transmitted by leafhoppers.

### Mastrevirus

Mastreviruses infect either monocots or dicots, and are transmitted by various leafhopper species [8]. Of the >30 species, members of the species *Maize streak virus* and *Wheat dwarf virus* are the best studied.

### Eragrovirus

This genus has one species, *Eragrostis curvula streak virus* [9].

### Grablovirus

This genus has one species, *Grapevine red blotch virus* [6].

### Topocuvirus

Isolates of the single species in this genus, *Tomato pseudo-curly top virus*, are transmitted by a treehopper [10].

### Turncurtovirus

*Turnip curly top virus* is the only species [11]. All isolates of this leafhopper-transmitted virus have been recovered from the dicot plants *Brassica rapa* or *Raphanus sativus* in Iran.

## Resources

Full ICTV Online (10th) Report: www.ictv.global/report/geminiviridae.
